# From the International Pediatric Sepsis Conference 2005 to the Sepsis-3 Consensus

**DOI:** 10.5935/0103-507X.20180005

**Published:** 2018

**Authors:** Daniela Carla de Souza, Marcelo Barciela Brandão, Jefferson Pedro Piva

**Affiliations:** 1 Pediatric Intensive Care Unit, Hospital Universitário, Universidade de São Paulo - São Paulo (SP), Brazil.; 2 Pediatric Intensive Care Unit, Hospital Sírio-Libanês- São Paulo (SP), Brazil.; 3 Pediatric Intensive Care Unit, Hospital das Clínicas, Universidade Estadual de Campinas - Campinas (SP), Brazil.; 4 Pediatric Intensive Care Unit, Hospital Estadual Sumaré - Sumaré (SP), Brazil.; 5 Department of Pediatrics, Universidade Federal do Rio Grande do Sul - Porto Alegre (RS), Brazil.; 6 Emergency Service and Pediatric Intensive Medicine, Hospital de Clínicas de Porto Alegre, Universidade Federal do Rio Grande do Sul - Porto Alegre (RS), Brazil.

## INTRODUCTION

In the last decades, sepsis, due to an immune response, has been defined as a potentially fatal organ dysfunction resulting from a dysregulated organism response to an infectious insult, in which pro- and anti-inflammatory responses can coexist in the early phase of the disease and which, together with non-immunological mechanisms, decisively influence prognosis and evolution.^(1-4)^ However, sepsis has complex pathophysiology and a varied and nonspecific clinical presentation, affecting heterogeneous groups of people; therefore, a simple and objective definition is not easy.^(2,5)^

Despite all efforts, severe sepsis and septic shock remain major causes of death in children, especially in developing and underdeveloped countries.^(6-11)^

In the SPROUT (Sepsis Prevalence, Outcomes, and Therapies) study in 2015, involving 7,000 children admitted to 128 pediatric intensive care units (ICUs) from 26 countries, the prevalence of severe sepsis was 8.2%, with wide variation between the continents: 6.2% in Europe and 23.1% in Africa (p < 0.001).^(12)^ In-hospital mortality due to severe sepsis was 25%, i.e., twice as high as the mortality rates reported in other studies.^(7,11-13)^ In another analysis of this database, there was a weak agreement (43%) between the clinical diagnosis of severe sepsis performed by the attending physician and the diagnostic criteria defined by consensus.^(14)^ The clinical diagnosis was performed in a more liberal way, with lower laboratory confirmation, presenting lower mortality and lower multiple organ failure than the group with severe sepsis defined by the 2005 consensus.^(14,15)^ This same discordance was described in adult patients with sepsis; however, interestingly, the divergence did not influence treatment and decision making.^(16)^ It is imperative for sepsis and its various stages to be defined in a precise way and for these definitions to be applied in both daily care practice and the evaluation of clinical studies.^(17,18)^

Pediatric age-specific definitions were only established in 2005 at the International Pediatric Sepsis Consensus Conference (IPSCC)^(13)^ and were based on various existing pediatric definitions, scores on organ dysfunction, and concepts of systemic inflammatory response syndrome (SIRS) and sepsis for the adult population. The task-force stated that the definitions represented an "instrument under construction" that still needed further refinement and improvement. Although not designed to improve the early diagnosis of bedside disease and to allow immediate therapeutic intervention, the definitions of sepsis proposed by the IPSCC have been used in daily clinical practice in pediatric ICUs around the world^(6-8,11-13,18,19)^ and formed the basis for the development of guidelines for the treatment of pediatric sepsis.^(19)^

In the last decade, several studies have demonstrated limitations of the definitions contained in the IPSCC,^(15)^ among which the following stand out.

**Imprecision:** Studies that adopted the definitions proposed by the 2005 consensus showed wide variations in prevalence (1 to 27%) and mortality (5 to 35%) due to severe sepsis in children.^(7,9,11,12,20-24)^ Part of this variability is due to the dynamic character of sepsis, in which the delimitations of disease stages may be tenuous. In this consensus, definitions of severe sepsis and septic shock (sepsis with cardiovascular dysfunction) describe the same stage of the disease, making the patient's classification in the severe sepsis or septic shock category dependent on the individual judgment of the attending physician.

**Applicability:** In several studies, difficulties encountered in applying the definitions in scenarios with limitations of resources are identified.^(8-12,23)^ The definitions of sepsis, severe sepsis, septic shock, and organ dysfunction require the laboratory tests, which are often not available or are difficult to obtain, delaying diagnosis and treatment.

**Low sensitivity and lack of agreement with clinical diagnosis:** Despite being in use for more than a decade, the definitions of the 2005 consensus have not been validated, with the aim of verifying their accuracy and applicability in different regions. Perhaps this lack of verification is the motivation for clinicians not to adhere to consensus definitions and to prefer to use their own or adapted criteria for defining sepsis and its stages.^(10,14)^ Some studies have shown that consensus definitions would be less sensitive in identifying suspected cases of sepsis than the clinician's diagnosis at the bedside.^(10,14)^ In one North American pediatric ICU, it was observed that one-third of the children with a clinical diagnosis of sepsis were not identified by IPSCC criteria and that only one-quarter were identified by all criteria.^(25)^ In the analysis of the SPROUT study database,^(12)^ it was observed that the results of sepsis studies that use the IPSCC diagnostic criteria cannot be applied to approximately one-third of the children diagnosed with sepsis hospitalized in pediatric ICUs. Furthermore, in approximately half of the patients identified as having severe sepsis by the IPSCC criteria and therefore eligible for participation in clinical trials, the diagnosis was not corroborated by the pediatric intensivist physician during their daily practice in the pediatric ICU. In this study, it is evident that the patients identified through the IPSCC criteria (more restricted definitions) would be in more advanced stages of sepsis, presenting more organ dysfunction, a greater frequency of chronic pathologies, and higher mortality, contrary to the principles of the Surviving Sepsis Campaign (SSC)^(26)^ and the American College of Critical Care Medicine/Pediatric Advanced Life Support (ACCM/PALS),^(27)^ for early diagnosis and rapid intervention.

In view of the above, there is no doubt that the definitions contained in the IPSCC criteria^(15)^ do not meet the wishes of clinicians or researchers.^(17)^ To overcome this challenge, sepsis was included in an urgent research agenda in Pediatric Intensive Medicine.^(17,28)^ In parallel, the definitions for adults have recently been published, and the possibility of "extending or adapting" these criteria for children has been proposed. In this sense, it is worth mentioning that Medicine is starting a new stage, conceptualized as "Precision Medicine". This concept is modeled on oncology, in which, based on an accurate diagnosis, it is possible for the physician to take an appropriate course for a specific situation.^(29)^ With this perspective, we analyze the limitations of using the definitions of the Third International Consensus Definitions for Sepsis and Septic Shock (Sepsis-3) for children:^(3,4)^

**Databases for extraction of variables:** The new definitions were based on retrospective analyzes of three databases of adult patients with sepsis in the United States and Germany. Of course, no pediatric patients were included nor were infectious diseases prevalent in other regions of the world, with a high prevalence of childhood sepsis (India, Asia, Africa, Latin America, among others).^(8,30)^

**Sensitivity and specificity:** The new definitions disregard SIRS presence, reducing sensitivity in favor of a possible increase in diagnostic specificity. In pediatrics, this loss of sensitivity would mean an expressive loss of diagnoses and, consequently, thousands of deaths without proper treatment in the early disease stages. Several studies have shown a high association of SIRS and sepsis in hospitalized children (~85%) and are sensitive for identifying children who progress to death, even with low specificity (15%). Differently from adults, for whom two or more SIRS criteria during the first 24 hours of ICU stay define severe sepsis with moderate sensitivity, it is observed that children hospitalized with SIRS criteria are at high risk of developing sepsis.^(10,31-33)^ Therefore, the inclusion of SIRS in the concept of sepsis in pediatrics meets at least one screening strategy aimed at early diagnosis and treatment.

**Organ dysfunction:** There is no doubt that the presence of organ dysfunction is also relevant in the context of pediatric sepsis.^(34)^ However, the Sequential Organ Failure Assessment (SOFA) score proposed for defining sepsis in adults has not been validated for children. In pediatrics, several scores were developed with the objective of predicting mortality in critically ill children admitted to the pediatric ICU. These scores were developed and validated in both developed and developing countries. Not only organ failure but especially the progression or the appearance of new organ failure has been associated with higher mortality.^(34)^ The use of scores such as the Performance of the Pediatric Logistic Organ Dysfunction (PELOD-2), which is the closest to the SOFA score applied in adults, has not been prospectively validated in children with sepsis admitted to the pediatric ICU and is not an instrument used in emergency services and hospitalization units, where there are many children with sepsis. Recently, a pediatric adaptation of the SOFA was evaluated retrospectively in a population of 6,300 children and adolescents with sepsis and septic shock admitted over seven years (2009-2016).^(35)^ Both the modified SOFA and PELOD-2 have good capacity to predict the mortality of groups of patients with sepsis and septic shock; however, their individual applicability to identify (sensitivity) and to confirm (specificity) cases has yet to be confirmed.^(35-39)^ Therefore, in pediatric sepsis, organ failure seems to be a useful variable in the follow-up, with some predictive specificity for mortality. However, with this perspective, its inclusion in the definition/identification of sepsis would not be justified.

**Hyperlactatemia:** Although some pediatric studies demonstrate that the initial increase of serum lactate would be a marker of severity and that its decrease would be associated with good response to therapy, it should be noted that several other factors may affect this increase or decrease. To date, there is no consistent demonstration that lactate is a marker of septic shock with acceptable accuracy in the definition of sepsis and its evolutionary stages nor as a therapeutic guide in these situations.^(18,19)^ Therefore, by adopting the Sepsis-3 criteria in the pediatric population, we would lose a reasonable portion of children with clinically established septic shock and with lactate levels below the definition threshold.

**Hemodynamic profile:** Children and adults differ in relation to the hemodynamic profile of septic shock, its clinical presentation, the presence and type of comorbidities, and the immune response to an infection. While hypotension is an early sign of shock in adults, it is a late sign in children, when they are already near collapse. If the presence of hypotension is included in the definition of pediatric septic shock, we will identify only those with advanced stages of the disease.

## CONCLUSION

Sepsis in children still presents a great challenge, with high incidence and high mortality rates. Ten years after the first consensus conference that defined sepsis in the pediatric population, we still look for sensitive and specific definitions for sepsis and its different stages.^(17,28)^

Current definitions are not accurate enough to be used by clinicians at the bedside, where early diagnosis and treatment are needed. In addition, current definitions include laboratory test results, many of which are not routinely available in resource-constrained scenarios.

However, we cannot repeat previous mistakes, such as adapting adult definitions for children, excluding systemic inflammatory response syndrome criteria from the definitions of sepsis for the pediatric population without judicious evaluation of its usefulness and including scores of organ dysfunction and screening for sepsis without extensive discussion and validation in different scenarios. Thus, there is no justification for Sepsis-3 to be incorporated in the context of pediatric sepsis. Instead, we must pursue precise definitions for each of the steps, which are easy to apply and guide the treatment at that particular stage.^(17,28,29)^

In the elaboration of the new definitions of sepsis for the pediatric population, it should be considered that a good portion of sepsis deaths in children occur in the early stage of the disease during the first 24 hours after admission to the pediatric ICU and even before admission to the ICU.^(18,36,37)^ Protocol education and implementation programs (e.g., ACCM/PALS) have been effective in reducing mortality. Therefore, the challenge in sepsis is not focused on its treatment but on its precise diagnosis, which should be based on clinical data and should use clinical screening instruments with applicability in the various scenarios, including regions with limited resources.
